# Balancing Time for Health Behaviors: Associations of Time Perspective With Physical Activity and Weight Management in Older Adults

**DOI:** 10.1177/08901171241242546

**Published:** 2024-04-02

**Authors:** Paul A. Davis, Michael Trotter, Elisabeth Åström, Michael Rönnlund

**Affiliations:** 1 Department of Psychology, 8075 Umeå University, Sweden

**Keywords:** time perspective, exercise, health behavior, healthy aging, diet, nutrition

## Abstract

**Purpose:**

To examine associations between time perspective and health promotion behaviors of physical activity and weight management.

**Design:**

Quantitative cross-sectional.

**Setting:**

This study is part of the Betula project on aging, memory, and dementia in Northern Sweden.

**Subjects:**

417 older adults aged between 55 and 85 years.

**Measures:**

Swedish-Zimbardo Time Perspective Inventory; Physical Activity in the past year, past week, and in comparison with others of similar age; Weight Management = Body Mass Index (BMI; kg/m^2^).

**Results:**

After controlling for age, sex, and years of education, hierarchical linear regression indicated a Balanced Time Perspective was significantly associated with more physical activity in the past year (*P* = .04), the past week (*P* < .001), and in comparison with others (*P* < .01). Past Negative time perspective was associated with less physical activity in the past year (*P* = .03), and in comparison with others (*P* = .03). Present Fatalistic was associated with less physical activity during the past week (*P* = .03), and in comparison with others (*P* = .01). Present Hedonistic was associated with more physical activity the past week (*P* = .03), and in comparison with others (*P* = .03). Past Negative was associated with higher BMI (*P* = .02), and Future Negative were associated with lower BMI (*P* = .01). Taken collectively, greater positivity and flexibility across time perspectives was associated with more physical activity, whereas negative oriented time perspectives related with less physical activity and poorer weight management.

**Conclusion:**

Time perspective can be associated with health behaviors in older adults and have implications for health across the lifespan. Health promotion interventions may target older adults’ enjoyment of exercise and weight management in the present, rather than highlight potential negative health outcomes in the future.

## Introduction

Extensive research indicates engaging in health promotion behaviors such as physical activity to maintain and optimize physical and mental health in later life is of central importance.^1,2^ However, the WHO reports as many as 80% of adolescents and 27.5% of adults are insufficiently physically active.^3^ In the United States of America these numbers increase where it is approximated that 76% of adults undertake insufficient physical activity and 42% possess a Body Mass Index (BMI) meeting the criteria for obesity (>30 kg/m^2^).^4^ The most common barrier impeding physical activity across the adult lifespan and into older age (eg, >60 years) is reported as a perceived lack of time.^5,6^ As such, increased understanding of the underpinning psychological factors influencing the perceived lack of time (as opposed to actual time) for physical activity is required.

Central to the experience of psychological time are individuals’ perceptions relative to temporal frames associated with the past, present, and future, which are collectively referred to as time perspective. Zimbardo and Boyd^7^ conceptualized time perspective into a five-factor model: Past Positive; Past Negative; Present Hedonistic; Present Fatalistic; and Future. More recently Carelli et al^8^ differentiated Future into Future Positive and Future Negative to account for worry and negative anticipation of the future and formed a six-factor model. Subsequent studies of older adults using the six-factor model have found associations between Future Negative and health related outcomes such as poor sleep quality,^9^ depressive symptoms,^10^ and stress^11^ although there is a lack of research examining potential links between physical activity and weight management in relation to Future Negative time perspective in late adulthood.

Research indicates an individual’s time perspective is often biased toward a particular temporal category with a direct influence on their cognitions, emotions, and behaviors.^12^ In relation to health promotion, negative biases in time perspective can degrade individuals’ intentions, planning, experience, and recall of physical activity^13^ as well as healthy eating habits.^14^ In contrast, a Balanced Time Perspective (BTP; ie, an optimal focus across each time perspective^9,15^) has been associated with better physical and psychological health,^16^ greater confidence and optimism in goal pursuit,^17^ and more effective coping with stress.^18^

Previous research examining associations between time perspective and health behaviors notes a positive relationship between Future time perspective and the intention to eat healthy,^19^ as well as promotion of weight management and physical activity in support of the treatment and management of recently diagnosed health conditions (eg, diabetes).^20^ However, a more recent study of the physical activity-time perspective relationship^21^ indicated cancer patients with a Present Fatalistic orientation scored lower on a measure of intention to exercise. In a sample taken from the general population, Griva et al^14^ observed only Future and Present Hedonistic dimensions of time perspective were associated with exercise behavior, although past negative and present fatalistic were related to higher BMI. Taken together these findings suggest the full spectrum of time perspectives warrants investigation both independently as well as in relation to each other (ie, balance vs deviation) when exploring associations with health promotion in later adulthood.

A present focused time perspective can be negatively associated with healthy behavioral practices,^22^ this is a challenge that is particularly relevant for older adults as potential future benefits of positive health behaviours may be perceived to be limited due to a shorter remaining lifetime. Research examining the relationship between thoughts of the future and exercise with older adults notes that a positive view of aging is associated with greater physical activity regardless of current health status.^23^ This finding lends support to healthy aging initiatives targeting physical activity as engaging in low intensity exercise (eg, walking) has been shown to promote functional ability^2^ and lower BMI in older adults.^24^

The health indicator of BMI has been noted across multiple studies examining associations between time perspective and health behaviors. A meta-analysis by Sweeney and Culcea^25^ comprised of 36 studies highlighted a significant association between future time perspective and healthier BMI. Further research has identified that both future and present time perspectives can indirectly predict BMI through changes in self-control (eg, improving food choices, limiting portion size). Specifically, a positive future time perspective predicts higher self-control and healthier BMI; whilst a more present time perspective has been found to predict lower self-control and higher BMI.^26^ Self-regulation has been noted as a central component in a recent meta-analysis of 378 studies examining associations between time perspective and meaningful outcomes across many domains of life including health and wellbeing.^27^ Engaging in physical activity can be regarded in multiple ways within a self-regulation framework when examining the role of time perspective in health promotion. Specifically, if an older adult undertakes the health protective behavior of exercising this can be considered a goal-directed behavior if the aim is weight management (eg, BMI <30 kg/m^2^); alternatively, if the goal is to meet the recommended levels of physical activity (eg, 150 minutes/week of moderate intensity exercise) undertaking exercise is a targeted outcome. As such, undertaking research examining both physical activity and weight management appears to be justified in attempts to increase understanding of the role of time perspective in healthy aging.

Considering the links between a range of important aspects of health and the noted promotion and treatment effects of physical activity and weight management,^24^ comprehensive examination of the full range of time perspectives is clearly warranted. Further, research examining the relationship between time perspective and health behaviors has not considered how a BTP may influence older adults’ physical activity and weight management (ie, BMI). As such, this study has two aims: (1) to determine if a deviation from a BTP predicts physical activity and BMI; and (2) to determine how each of the individual time perspective factors independently predict physical activity and BMI in later adulthood.

## Method

### Participants

The sample consisted of 417 participants, 217 females and 200 males, aged between 55 and 85 years (*M* = 64.98, *SD* = 7.59) from the Betula prospective study (see Ref. 28). Participants provided informed consent prior to commencing voluntary involvement in the study. The study has been undertaken under the approval from the Swedish Ethics Review Authority. Data from test wave six were included as this was the only wave to include a measure of time perspective.^9^

### Measures

*Time Perspective* was measured using the Swedish version of the S-ZTPI. The S-ZTPI has 64 items and six factors: Future Positive; Future Negative; Present Hedonistic; Present Fatalistic; Past Positive; and Past Negative. Items are measured on a 5-point Likert scale with anchors ranging from 1 = very uncharacteristic to 5 = very characteristic. Previous research using the S-ZTPI has demonstrated acceptable reliability and validity.^7^

*Deviation from Balanced Time Perspective* (DBTP) was operationalized using the method first proposed by Stolarski et al,^29^ and later extended by Rönnlund et al^11^ to also include scores on the Future Positive versus Negative subscales. The method summarizes the deviations from optimal ZTPI scores across all six subscales. Recently Jankowski et al^30^ proposed a revised formula (DBTP-r) that was adopted in the present study and extended to the S-ZTPI:

$$\sqrt{{\left(1-ePN\right)}^{2}+{\left(5-ePP\right)}^{2}+{\left(1-ePF\right)}^{2}+{\left(3.4-ePH\right)}^{2}+{\left(5-eFP\right)}^{2}}+{\left(1-eFN\right)}^{2}$$

The values (1-5) represent the optimal score for each ZTPI subscale (eg, PN = 1, PP = 5). Expressions with postscript *e* denotes the observed (empirical) mean score for the corresponding subscale (eg, *ePN* = mean for the Past Negative subscale).

*Physical activity* was measured with three single-item measures similar to previous research of physical activity and time perspective.^14^ Participants referenced their level of physical activity during leisure time for the past year, “How much have you moved and strained yourself physically in your leisure time in the last 12 months?” measured on a 4-point Likert scale: 1 = Sedentary leisure time (eg, reading, television, cinema, or other sedentary leisure activities); 2 = Moderate exercise during leisure time (eg, walking/cycling from work, heavy housework, gardening, fishing, table tennis, bowling); 3 = Moderately regular exercise during leisure time (eg, running, swimming, tennis, badminton, or other activity that makes you sweat); 4 = Regular exercise and training (eg, 30 minutes or more of running, swimming, playing tennis or badminton, aerobics, or similar at least 3 times per week). Participants also indicted how physically active they were relative to WHO^3^ recommendations of level of exercise intensity during the past week, “How much time do you spend in a typical week doing moderately strenuous activity that causes you to be warm?” on a 5-point Likert scale with anchors being: (4) = 5 hours a week or more; (3) = More than 3 hours but less than 5 hours a week; (2) = Between 1 and 3 hours a week; (1) = No more than one hour a week; (0) = Not at all. Finally, participants compared their physical activity relative to others of a similar age, “Compared with the other people your age how physically active do you think you are?” on a 5-point Likert scale with the following anchors: (5) = much more; (4) = somewhat more; (3) = same; (2) = somewhat less; (1) = much less.

*Weight Management* was measured via Body Mass Index (BMI). Participants' weight and height were recorded by a research assistant. BMI was calculated as: BMI = *kg/m*^2^ (kg = weight; m = height^31^).

*Statistical Analyses* were conducted with SPSS version 28. Data were screened for outliers; incomplete data were removed from the dataset, and descriptive statistics were run for all variables. Pearson correlations were calculated to determine associations between all study variables. Hierarchical linear regressions examined DBTP as a predictor of physical activity and weight management. DBTP was entered as the predictor variable and the physical activity and BMI variables as the outcome variables, demographic variables (ie, age, sex, years of education) were entered in the first block and DBTP was entered in the second block. Hierarchical multiple regressions examined the predictive nature of the S-ZTPI subscales on physical activity and weight management. S-ZTPI subscales were entered as the predictor variable, with physical activity and BMI variables as the outcome variables. Demographic variables were included in block one and S-ZTPI subscales added in block two. Prior to testing the research questions the independent variables were examined for collinearity. All variables had variance inflation under 2.1. All factors had collinearity tolerance above an acceptable level (>.10).

## Results

Means, standard deviations, and correlations for age, sex, years of education, BMI, physical activity, DBTP, and time perspective subscales are displayed in Table 1. The correlational analyses revealed that age was weakly negatively correlated with leisure time physical activity in the past year. Age was weakly positively correlated with DBTP, Future Negative, Past Positive, and Past Negative; age was moderately positively correlated with Present Fatalistic. Years of education was weakly positively correlated with leisure time physical activity in the past year and with Future Positive. Years of education was moderately negatively correlated with Present Fatalistic and weakly negatively correlated with DBTP. Leisure time physical activity in the past year was strongly positively correlated with comparative physical activity and moderately positively correlated with physical activity per week. Leisure time physical activity in the past year was weakly negatively correlated with Present Fatalistic, Past Negative, and DBTP. Physical activity time per week was moderately positively correlated with comparative physical activity. Physical activity time per week was weakly negatively correlated with Future Negative, Present Fatalistic, Past Negative, and DBTP. Comparative physical activity was weakly positively correlated with Future Positive, and weakly negatively correlated with Present Fatalistic, Past Negative, and DBTP. BMI was moderately negatively correlated with comparative physical activity and weakly negatively correlated with leisure physical activity in the past year, and physical activity time per week.

**Table 1.** Descriptive Statistics and Pearson Moment Correlations for all Study Variables.

Variable	1	2	3	4	5	6	7	8	9	10	11	12	13	14
1. Age														
2. Education	−.41**													
3. Sex	<−.01	−.09												
4. PA PY	−.19**	.17**	.02											
5. PA PW	−.05	.02	−.12*	.41**										
6. Comp PA	.02	.05	.07	.51**	.45**									
7. BMI	−.06	−.01	<−.01	−.12*	−.19**	−.32**								
8. FN	.12*	−.04	−.09	−.09	−.10*	−.03	−.05							
9. FP	−.02	.16**	.06	.05	−.02	.12*	−.02	.32**						
10. PF	.28**	−.32**	−.11*	−.17**	−.10*	−.15**	.02	.43**	−.12*					
11. PH	.03	.03	−.05	.02	.08	.04	.08	.17**	−.05	.37**				
12. PN	.18**	−.08	.02	−.15**	−.16**	−.11*	.07	.62**	.15**	.38**	.14**			
13. PP	.10*	<.01	−.04	<−.01	.10*	.05	−.05	−.04	.14**	.09	.26**	−.26**		
14. DBTP	.17**	−.23**	−.03	−.11*	−.18**	−.14**	<-.01	.47**	−.20**	.53**	−.20**	.60**	−.54**	
M	64.98	12.85	1.48	2.54	2.71	3.29	26.76	2.51	3.27	2.49	2.91	2.25	3.59	2.32
SD	7.59	4.21	.50	.87	1.30	.97	3.71	.54	.42	.53	.43	.59	.52	.64

Note. **significant at the .01 level (2-tailed), *significant at the .05 level (2 tailed).

Results of the hierarchical linear regressions for the analysis between DBTP and leisure time physical activity in the past year and physical activity time per week are reported in Table 2, comparative physical activity and BMI are reported in Table 3. A statistically significant negative association was observed between leisure time physical activity in the past year and DBTP, such that participants with greater DBTP reported that they were less physically active in the last 12 months. A statistically significant negative association was observed wherein participants with greater DBTP reported they were less physically active in the past week. A statistically significant negative association was observed between comparative physical activity and DBTP, specifically participants with greater DBTP reported they were less physically active compared to others of their age. DBTP was not found to predict BMI.

**Table 2.** Physical Activity Past Year and Past Week Hierarchical Multiple Regression Results.

		PA time past year						PA time per week					
	Model	1			2			1			2		
	Variable	β	*t*	*P*	β	*t*	*P*	β	*t*	*P*	β	*t*	*P*
Demo	Age	−.14	−2.61	.01	−.13	−2.37	.02	−.05	−.95	.34	−.03	−.55	.58
	Sex	.12	2.23	.03	.10	1.87	.06	−.01	−.24	.81	−.05	−.83	.41
	Years Edu	.02	.41	.69	.01	.23	.82	−.13	−2.58	.01	−.14	−2.91	<.01
S-ZTPI	DBTP				−.10	−2.04	.04				−.18	−3.62	<.001
	*R* ^2^		.05			.06			.02			.05	
	Adj. *R*^2^		.04			.05			.01			.04	
	Δ*R*^2^		.05			.01			.02			.03	
	*F*_*change*_		6.67			4.15			2.49			13.08	

Note. PA = Physical activity.

**Table 3.** Comparative Physical Activity and BMI Hierarchical Multiple Regression Results.

		Comparative PA						BMI					
	Model	1			2			1			2		
	Variable	β	*T*	*P*	β	*t*	*P*	β	*t*	*P*	β	*t*	*P*
Demo	Age	.05	.84	.40	.06	1.16	.25	−.08	−1.48	.14	−.08	−1.54	.13
	Sex	.08	1.40	.16	.05	.92	.36	−.01	−.25	.80	−.01	−.20	.85
	Years Edu	.07	1.36	.17	.06	1.13	.26	−.05	−.87	.39	−.04	−.75	.45
S-ZTPI	DBTP				−.15	−2.86	<.01				.03	.63	.53
	*R* ^2^		.01			.03			.01			.01	
	Adj. *R*^2^		<.01			.02			<-.01			<-.01	
	Δ*R*^2^		.01			.02			.01			<.01	
	*F*_*change*_		1.18			8.16			.76			.39	

Note. PA = Physical activity.

Results of the hierarchical linear regressions for the analysis of the S-ZTPI subscales and leisure time physical activity in the past year and physical activity time per week are reported in Table 4, comparative physical activity and BMI are reported in Table 3. A statistically significant negative association was observed between participants with greater levels of Past Negative reporting less time being physically active over the previous year. A statistically significant negative association was observed with participants who reported greater levels of Present Fatalistic being less physically active during the past week. A statistically significant positive association was observed for participants who reported greater levels of Present Hedonistic spending more time being physically active during the previous week. Statistically significant negative associations were observed for participants who reported greater levels of Present Fatalistic and Past Negative indicating they perceived themselves to be less physically active compared to others their age. A statistically significant positive association was observed for participants who reported greater levels of Present Hedonistic being more physically active compared to others their age. A statistically significant positive association was observed with participants who reported greater levels of Past Negative measuring higher BMI. Conversely, Future Negative was found to predict lower BMI; no statistically significant associations were observed for the other subscales of the S-ZTPI Table 5.

**Table 4.** Physical Activity Past Year and Past Week Hierarchical Multiple Regression Results.

		PA time past year						PA time per week					
	Model	1			2			1			2		
	Variable	B	*T*	*P*	β	*t*	*P*	β	*t*	*P*	B	*t*	*P*
Demo	Age	−.14	−2.61	.01	−.10	−1.82	.07	−.05	−.95	.34	−.02	−.29	.77
	Sex	.02	.41	.69	.01	.25	.80	−.13	−2.58	.01	−.14	−2.72	.01
	Years Edu	.12	2.22	.03	.09	1.57	.12	−.01	−.24	.81	−.05	−.97	.33
S-ZTPI	FN				.02	.21	.83				<.01	.06	.95
	FP				.06	1.09	.28				−.02	−.27	.78
	PF				−.08	−1.17	.24				−.14	−2.22	.03
	PH				.09	1.73	.09				.12	2.15	.03
	PN				−.15	−2.23	.03				−.11	−1.68	.09
	PP				−.06	−1.10	.27				.06	1.02	.31
	*R* ^2^		.05			.08			.02			.07	
	Adj. *R*^2^		.04			.05			.01			.04	
	Δ*R*^2^		.05			.03			.02			.05	
	*F*_*change*_		6.67			2.01			2.49			3.37	

Note. PA = Physical activity.

**Table 5.** Comparative Physical Activity and BMI Hierarchical Multiple Regression Results.

		Comparative PA						BMI					
	Model	1			2			1			2		
	Variable	β	*t*	*P*	β	*t*	*P*	β	*T*	*P*	B	*t*	*P*
Demo	Age	.05	.84	.40	.09	1.59	.11	−.08	−1.48	.14	−.09	−1.65	.10
	Sex	.07	1.36	.17	.05	1.07	.28	−.01	−.25	.80	−.03	−.54	.59
	Years Edu	.08	1.40	.16	.01	.15	.88	−.05	−.87	.39	−.05	−.82	.41
S-ZTPI	FN				.10	1.38	.17				−.18	−2.64	.01
	FP				.09	1.61	.11				.04	.69	.49
	PF				−.18	−2.83	.01				.03	.44	.66
	PH				.12	2.22	.03				.10	1.88	.06
	PN				−.15	−2.21	.03				.16	2.29	.02
	PP				−.02	−.38	.70				−.04	−.74	.46
	*R* ^2^		.01			.06			.01			.04	
	Adj. *R*^2^		<.01			.04			<-.01			.02	
	Δ*R*^2^		.01			.05			.01			.03	
	*F*_*change*_		1.78			3.71			.76			2.27	

Note. PA = Physical activity.

## Discussion

The present study examines the role of time perspective in promotion of the health behaviors relating to engaging in physical activity and weight management. In a sample of older adults, we found that BTP was related with perceptions of being more physically active over the past year and past week, as well as in comparison to others of a similar age. Previous studies have highlighted associations between BTP and promotion of subjective wellbeing,^8^ positive mental health,^32^ as well as cognition.^33^ Multiple explanations have been advanced in attempts to outline the ‘pathways’ by which time perspectives may be associated with wellbeing.^34^ For example, it is posited that ‘temporal flexibility’ can assist individuals to effectively regulate emotions, decrease tension, as well as increase energy and happiness.^35^ The temporal flexibility of BTP can facilitate self-regulation^27^ and behavioral choices (eg, leisure activities, health behaviors, retirement planning) which promote a healthy lifestyle and has implications for optimal aging.^11,36^ The findings of the present study contribute to previous research as we observed associations between BTP and the health behavior choice of engaging in physical activity during leisure time. As such, this study highlights a potential link between physical activity and temporal flexibility for health promotion in older adults.

The second aim of the study was to examine independent relationships that may exist between the constituent aspects of time perspective, physical activity, and weight management. We found individuals with Past Negative time perspective perceived themselves to engage in less physical activity in the past year and past week, as well as in comparison with peers of a similar age. Present Fatalistic individuals also reported themselves to undertake less physical activity in the past week and in comparison with others. Previous research with cancer patients similarly noted a negative relationship between Present Fatalistic time perspective and intentions to exercise,^21^ the present study extends this finding to generally healthy older adults. Further, the negative influence of Present Fatalistic and Past Negative time perspectives on measures of health is noted across a range of studies^14,37^ and has been linked with physical components of pain.^38^ Previous studies note associations between levels of physical activity and pain in older adults^39^; as such individuals with Present Fatalistic and Past Negative time perceptions who perceived themselves to be less physically active, may have concerns about experiencing exercise-related pain. Future research is warranted to examine underlying factors (eg, previous injuries) that inform older adults’ perceptions and decision-making regarding physical activity for health promotion.

In the present study, Past Negative time perspective was found to be associated with older adults’ BMI. Previous research with adults between the ages of 18-56 found a similar relationship wherein those with a higher BMI reported greater Past Negative^14^; in the present study we extended this research by considering Future Negative and examining associations between time perspective and weight management in older adults. Further, it was noted that BMI scores were negatively associated with Future Negative, this result supports the findings of a meta-analysis^25^ wherein Future time perspective is noted to be associated with a healthier BMI. However previous studies have not differentiated between Future Negative and Positive time perspectives; this study is the first to indicate the association with BMI is consistent across both time perspectives. Extensive research and health promotion interventions have targeted Future time perspective with the aim of promoting health behaviors,^40^ prospective studies would be well served to differentiate between Future Positive and Negative time perspectives to promote greater sustainability of health choices wherein individuals’ attempts at self-regulation align with pursuit of their best possible selves rather than avoidance of negative health outcomes.^27^

Older adults with higher levels of Present Hedonistic perceived themselves to be more physically active, this finding is in line with previous research of time perspective and health-related behaviors.^14,41^ A potential explanation may be that individuals who have increased their physical activity over time consider exercise to be pleasurable and chose to undertake physical activity to promote enjoyment in the present. This finding relates with research examining affective responses to exercise^42^ and can guide health promotion interventions aiming to optimize temporal aspects of exercise related affect. Specifically, the recall of affective responses to exercise has been noted to be influenced by the duration of time elapsed since previous exercise activity^43^ with implications for forecasted pleasure^44,45^ as well as affective responses during physical activity.^46^ Previous interventions have addressed time perspective to increase physical activity with young adults,^22^ although the focus has predominantly centered on future time perspective and long-term health benefits. Future research examining the efficacy of interventions targeting future time perspective in older adults is warranted to determine if perceptions of limited lifespan remaining diminishes the evaluation of health benefits to be derived from physical activity.^47^

Time perspective was previously suggested to be a stable individual difference,^7^ however more recent experimental studies have demonstrated that they can be augmented.^22,27^ Modifying older adults’ time perspective, or increasing temporal flexibility, could be a promising avenue for health promotion interventions designed to increase physical activity and weight management. Intervention-based studies may seek to target older adults’ present time perspectives and increase enjoyment and positive evaluations of exercise related affect to promote adherence and sustainable physical activity behaviors.^48^

Although this study offers novel insight into the relationship between time perspective and the promotion of physical activity and weight management, it does include several limitations that warrant attention. First, despite the Betula database containing longitudinal data, the time perspective measure was only recorded at one time point, subsequently restricting the present study to the use of a cross-sectional research design. As such we are unable to infer causal conclusions from the findings. Unfortunately, this is a widely acknowledged shortcoming as longitudinal research is called for in many discussion sections of studies examining time perspective.^49^ Age-related changes are noted to relate to time perspective across the lifespan,^50^ considering the sample in the present study older individuals are often more negative about future opportunities and shift their attention to emotion-related goals rather than work-related pursuit. Future studies would be well served by using repeated measures and longitudinal research design to facilitate investigation of how fluctuations in time perspective might influence emotions underpinning health behaviors associated with physical activity and weight management over time.

It also warrants noting that numerous studies have identified biases in self-reports of physical activity, diet, and BMI^51,52^ due to socially desirable responding, subjective interpretations, or inaccurate recall on the part of the respondents. Future research could be enhanced by integrating multiple methods of assessment into research designs, recent advances in the use of wearable technology (eg, activity trackers) can offer more insightful objective measures of physical activity and factors influencing weight management.^53^

The present study was undertaken within the geographical area of Northern Sweden using a sample that was determined to be representative of the target population at time of the study^9^ whilst controlling for the variables of age, sex, and years of education in the data analyses. That said, the study’s findings may be somewhat limited in terms of their generalizability to populations comprised of more diverse cultures. For example, results of a meta-analysis indicates that associations between future time perspective and health behaviors are more pronounced in individualistic rather than collectivistic cultures.^50^ Older adults in individualistic countries are more likely to be expected to take care of themselves; as such, to facilitate healthy aging individuals may invest in healthy behaviors (eg, exercise, healthy eating) across the lifespan. Future, studies with more diverse samples across varying levels of individualism/collectivism are suggested to consider factors relating to socioeconomic status, mental health conditions, or specific health conditions that could affect physical activity and weight management.

In consideration of the complexity of the factors guiding health behaviors, influences beyond the scope of the present study may have impacted upon the findings we have reported. For example, an individual’s motivation, self-efficacy, and social support have been noted to underlie physical activity.^54^ Additionally, although BMI is the traditional metric used to measure adiposity due to it being a quick and inexpensive method of assessment, it does have limitations relating to body composition and fat distribution^55^ that can fail to reflect individuals’ weight management and healthy eating behaviors (eg, portion size). Although BMI is widely used in studies of time perspective and health behaviors,^25^ caution is advised in the interpretation and application of the findings reported in these studies; future research may endeavor to use a variety of measures when investigating time perspective and health promotion. Further research may examine individuals’ appraisals of health benefits that can be derived from physical activity, as this can underpin the influence of future time perspective on exercise intentions.^47^ Examining the related concept of time orientation and its cognitive emphasis relating to consideration of future consequences^48^ could elucidate the trade-off between long- and short-term behavioural outcomes influencing physical activity and weight management in older adults.

The findings of this study lend support to health promotion initiatives seeking to increase physical activity and healthy weight management. Health promotion interventions may benefit from fostering temporal flexibility in relation to affective responses to exercise, this can optimize how individuals remember, anticipate, and experience optimal affect during physical activity. Further, older adults can be more motivated to engage in exercise and maintain a healthy weight for enjoyment in the present, rather than reinforce feelings of guilt associated with past behavior or highlight potential negative health outcomes in the future. Finally, to promote healthy aging future research may study how affective responses to exercise and eating are influenced by self-regulation and time perspective across the lifespan.

**“So What?”**

**What is Already Known on This Topic?**

• The most common barrier impeding physical activity across the adult lifespan and into older age (eg, >60 years) is reported as a perceived lack of time.
• Central to the experience of psychological time are individuals’ perceptions relative to temporal frames associated with the past, present, and future, which are collectively referred to as time perspective.

**What Does This Article Add?**

• This study notes a Balanced Time Perspective (ie, an optimal focus across time perspectives relating to past, present, and future) is associated with being more physically active.
• Time perspectives relating to negative evaluations of the past and present were associated with less physical activity and higher BMI.

**What are the Implications for Health Promotion Practice or Research?**

• Health promotion initiatives seeking to increase physical activity and weight management may aim to enhance enjoyment of exercise in the present as opposed to communicating health benefits that can be derived from physical activity in the future.
• Future research may study how affective responses to exercise and weight management are influenced by individual differences in time perspective across the lifespan to promote healthy aging.
